# Dexmedetomidine provides renoprotection against ischemia-reperfusion injury in mice

**DOI:** 10.1186/cc10283

**Published:** 2011-06-24

**Authors:** Jianteng Gu, Pamela Sun, Hailin Zhao, Helena R Watts, Robert D Sanders, Niccolo Terrando, Peiyuan Xia, Mervyn Maze, Daqing Ma

**Affiliations:** 1 Department of Anaesthetics, Pain Medicine and Intensive Care, Faculty of Medicine, Imperial College London, Chelsea and Westminster Campus, 369 Fulham Rd, London SW10 9NH, UK; 2 Department of Pharmacy, Southwest Hospital, Third Military Medical University, 30 Gaotanyan Road, Chongqing 400038, China; 3 Department of Anesthesia and Perioperative Care, UCSF, 521 Parnassus Avenue, San Francisco, CA 94143, USA; 4 Department of Anesthesiology, Hubei University of Medicine, Shiyan, Hubei 442000, P.R. China

## Abstract

**Introduction:**

Acute kidney injury following surgery incurs significant mortality with no proven preventative therapy. We investigated whether the α_2 _adrenoceptor agonist dexmedetomidine (Dex) provides protection against ischemia-reperfusion induced kidney injury *in vitro *and *in vivo*.

**Methods:**

*In vitro*, a stabilised cell line of human kidney proximal tubular cells (HK2) was exposed to culture medium deprived of oxygen and glucose. Dex decreased HK2 cell death in a dose-dependent manner, an effect attenuated by the α_2 _adrenoceptor antagonist atipamezole, and likely transduced by phosphatidylinositol 3-kinase (PI3K-Akt) signaling. *In vivo *C57BL/6J mice received Dex (25 μg/kg, intraperitoneal (i.p.)) 30 minutes before or after either bilateral renal pedicle clamping for 25 minutes or right renal pedicle clamping for 40 minutes and left nephrectomy.

**Results:**

Pre- or post-treatment with Dex provided cytoprotection, improved tubular architecture and function following renal ischemia. Consistent with this cytoprotection, dexmedetomidine reduced plasma high-mobility group protein B1 (HMGB-1) elevation when given prior to or after kidney ischemia-reperfusion; pretreatment also decreased toll-like receptor 4 (TLR4) expression in tubular cells. Dex treatment provided long-term functional renoprotection, and even increased survival following nephrectomy.

**Conclusions:**

Our data suggest that Dex likely activates cell survival signal pAKT *via *α_2 _adrenoceptors to reduce cell death and HMGB1 release and subsequently inhibits TLR4 signaling to provide reno-protection.

## Introduction

Perioperative acute kidney injury (AKI) is an abrupt deterioration of renal function that occurs as a complication of major cardiothoracic, vascular and transplant surgery [1-5]. In this setting AKI is associated with prolonged hospitalization and mortality rates as high as 60% [6,7]; including a 25-fold increase in mortality following cardiac valve surgery [7,8]. Furthermore, patients who sustain AKI and make a full recovery retain a higher risk of long-term mortality [9].

Among its diverse etiologic factors, ischemia-reperfusion injury (IRI) remains the foremost cause of perioperative AKI [10]. Following a transient deprivation of total or regional vascular supply to the kidney, restoration of blood flow inflicts continuous and severe damage in the post-ischemic renal parenchyma, characterized histopathologically as vascular, tubular, and inflammatory perturbations [11]. A growing body of evidence demonstrates that the TLR family, particularly TLR-4, plays the dominant role in mediating the deleterious effects in renal IRI [12,13]. In addition, damage-associated molecules such as HMGB-1 have been postulated as a TLR-4 ligand that drives the robust inflammatory response in post-ischemic kidney [14,15].

The current clinical management of perioperative AKI is supportive [16]; therefore, novel prophylactic (pre-insult therapy) and therapeutic (post-insult therapy) is required to reduce the burden of AKI in the perioperative period. The α_2 _adrenoceptor agonist dexmedetomidine exerts sedative, analgesic, hemodynamic stabilizing, anti-inflammatory and diuretic effects [17]. It is a highly potent α_2 _adrenergic agonist with a remarkable binding specificity for the α_2 _adrenoceptor. Novel organoprotective properties of dexmedetomidine have been explored in the brain, heart and renal injury [18-21]. Indeed α_2 _adrenoceptors are distributed widely in the renal proximal, distal tubules and peri-tubular vasculature. Clinically α_2 _adrenoceptor agonists enhance urine flow rate and perioperative renal function [22,23]; however, the underlying molecular mechanisms remain unknown. Animal studies have suggested that α_2 _adrenoceptor agonists are renoprotective as a class; their mechanism largely revolving around modulating vasoreactivity [21,24,25]. Herein we report that dexmedetomidine protects against IRI to the kidney in mice and that the mechanism is due to a decrease in the level of renal cell death and suppression in the HMGB-1-TLR-4 inflammatory circuit.

## Materials and methods

### Cell line

A stabilised cell line of kidney cells (HK2), derived from adult human kidney proximal tubular cells, was used in our experiments (European Cell Culture Collection, Salisbury, UK). Cells were cultured in RPMI 1640 medium, 1% L-glutamine 100 nM, 1% penicillin-streptomycin 100 U/ml, 5% fetal calf serum (Gibco, Invitrogen Ltd, Paisley, UK) in a humidified atmosphere containing 5% CO_2_. They were used soon after reaching 80% confluence.

### Cell treatments

Cell injury was provoked by oxygen glucose deprivation (OGD) as we reported previously [26]. Briefly, OGD solution (116 mM NaCl, 5.4 mM KCl, 0.8 mM MgSO_4_, 1.0 mM NaH_2_PO_4_, 26 mM NaHCO_3 _and 1.8 mM CaCl_2_; pH 7.4) was bubbled through with pure nitrogen gas for 15 minutes using sterile Drechsel bottles to remove oxygen from the solution. Cells were washed sequentially with warmed HEPES buffer solution (120 mM NaCl, 5.4 mM KCl, 0.8 mM MgCl_2_, 1.8 mM CaCl_2_, 15 mM anhydrous D-glucose and 20 mM HEPES) and warmed prepared OGD solution. The multi-well plates were then cultured with 1 ml of warmed OGD solution and incubated in air tight gas chambers for the indicated period exposed to 95% nitrogen, 5% CO_2 _at 37°C with or without dexmedetomidine (0.001 to 0.1 nM) (Orion Pharm (UK) Ltd, NewburyBerkshire, UK)+/- atipamezole (1 nM). After OGD treatment, cells were removed from the gas chamber, the OGD solution was replaced with warmed culture medium and placed in a humidified 5% CO_2 _incubator at 37°C for 24 hrs. Cell viability was assessed using an 3-(4,5-Dimethyl-2-thiazolyl)-2,5-diphenyl-2H-tetrazolium bromide (MTT, Merck KGaA, Darmstadt, Germany) assay [26].

Other cell cohorts were treated with dexmedetomidine (0.1 nM) in the absence of OGD with or without the highly selective inhibitors of PI3-Akt, LY294002 (50 μM), and mitogen-activated protein kinase (MAPK), PD98059 (50 μM), respectively.

### Animals

Ten-week-old male C57BL/6J mice weighing 20 to 25 g were housed in temperature and humidity-controlled cages with free access to sterile acidified water and irradiated food in a specific pathogen-free facility at Imperial College London. This study was approved by the ethics committee of Imperial College London and the UK Home Office (PPL: 70/6966) and all procedures were performed strictly under the United Kingdom Animals (Scientific Procedures) Act 1986.

### Renal ischemia-reperfusion injury

Dexmedetomidine (25 μg/kg, i.p., based on the previous organ protection studies [19,27]) was administered 30 minutes before or immediately after renal ischemia-reperfusion injury (rIRI). One cohort was treated with the α2 adrenoceptor antagonist atipamezole (250 μg/kg, i.p. [19,27]) prior to the administration of dexmedetomidine. The naive group and the rIRI group served as negative and positive controls, respectively. The animals were sacrificed 24 hr after rIRI. Kidneys were harvested for H&E and terminal deoxynucleotidyl transferase dUTP nick end labeling (TUNEL) staining. All assessments were made by an investigator who was blinded to the experimental protocols. rIRI was induced either by bilateral renal pedicle clamping for 25 minutes to produce moderate renal injury, or by right renal pedicle clamping for 40 minutes and left nephrectomy to produce life-threatening renal injury, under 1.5% isoflurane surgical anesthesia. Sham-operated mice had dissection as above, but with no occlusion of the renal vessel. The intra-abdominal temperature was maintained at 36 ± 0.1°C with a heating pad which was servo-adjusted by a temperature controller (Engineering Inc, Stamford, CT, USA) throughout the experiment. For survival experiments, mice were monitored on a daily basis with a scoring assay based on body weight, activity and general appearance as reported previously. Any animals that scored > 7 were euthanized. All animals received 0.5 ml saline i.p. injection per every 6 hrs for the first 24 hrs after experiments.

### Immunoblot

Proteins were extracted from treated HK2 cells lines or frozen kidney samples by cell disruption in cell lysis buffer (New England Biolabs, Hitchin, Hertfordshire, UK) and sonication with an ultrasonic probe, followed by centrifugation at 10,000 g for 10 minutes at 4°C. The supernatant was collected for Western blotting. Samples containing 30 μg of extracted protein, as determined by the Bradford protein assay (BioRad, Hemel Hempstead Hertfordshire, UK), were loaded on a NuPAGE 4 to 12% Bis-Tris gel (Invitrogen, Paisley, UK) for protein fractionation by electrophoresis and then electro-transferred to a nitrocellulose membrane (Hybond ECL; Amersham Biosciences, Little Chalfont, Buckinghamshire, UK). Blots were blocked with 5% non-fat dry milk in TBS (pH8.0, containing 0.1% Tween-20), and probed with appropriate antibodies (1:1,000 Akt, p-Akt and TLR4- all from followed Cell Signaling, Hitchin, Hertfordshire, UK; 1:100 TLR4- Santa Cruz, Wembley Middlesex, UK) by HRP-conjugated secondary antibodies (Cell Signaling) and visualisation with enhanced chemiluminescence (Cell Signaling). α-tubulin (1:2,000, Sigma, Dorset, UK) was used as internal control. Densitometry analysis were preformed and normalized with α-tubulin and then presented as percentage of control.

### Histologic score

The sum score was calculated from the analysis of 10 cortical tubules/cross-section stained with H&E (10 sections/kidney) by using a modified scoring system [28]; 0, no damage. 1, mild damage: rounded epithelial cells and dilated tubular lumen; 2, moderate damage: flattened epithelial cells, loss of nuclear staining and substantially dilated lumen; 3, severe damage: destroyed tubules with no nuclear staining of epithelial cells.

### Immunohistochemistry

The second l death of tubular epithelial cells was detected by *in situ *TUNEL assay Obiogene, Cambridge, UK) according to the manufacturer's instructions. The fixed cryostat sections were washed with PBS and then treated with proteinase K (20 μg/ml) at room temperature for 15 minutes. For positive controls, sections were treated with nuclease (R&D System, Abingdon, UK) at 37°C for 15 minutes. The sections were quenched in 3% hydrogen peroxide in PBS for five minutes. The quenched sections were labelled with TDT enzyme at 37°C for 1 hour in a humidified chamber and subsequently incubated with anti-digoxygenin conjugated to horseradish peroxidase for 30 minutes at room temperature. They were then stained with DiAminoBenzidine (DAB). The sum of the TUNEL^+ ^cells in an objective grid from 10 areas of randomly selected renal cortex was counted under a 40 × objective lens by an investigator who was blinded to the experimental protocol.

The other fixed cryostat sections were incubated with 3% hydrogen peroxide for 30 minutes to quench endogenous peroxidase activity. Sections were blocked for 30 minutes in wash buffer phosphate buffered saline tween-20 containing 3% goat serum and then incubated at 4°C overnight with goat anti-mouse TLR-4 (Santa Cruz Biotechnology, Wembley, Middlesex, UK) diluted 1:250 in PBS and 1% goat serum. After washing with PBST, sections were incubated with biotinylated donkey anti-goat IgG (Santa Cruz) diluted 1:250 in PBS and then labelled with VectaStain Elite ABC solution (Vector Labs, Orton Southgate, Peterborough, UK). After further washes in PBS, staining was developed with DiAminoBenzidine (DAB) with nickel. Slides were washed in PBS buffer, dH20 and then in 100% ethanol for 10 minutes and xylene for 10 minutes before covered with cover glass for micrograph taken.

### Plasma creatinine, urea and HMGB1

Both creatinine and urea were measured in 100 μl of plasma with an Olympus AU640 analyzer (Diamond Diagnostics, Watford, UK). Plasma HMGB1 was measured by ELISA according to the manufacturer's instruction (IBL International GmbH, Hamburg, Germany)

### Statistical analysis

Statistical comparison was by ANOVA followed by *post hoc *Student-Newman Keul's test where appropriate. Survival was analyzed by Kaplan-Meier test. A *P *< 0.05 was considered as statistically significant.

## Results

### Dexmedetomidine confers *in vitro *protection via Akt activation

**Figure 1:**
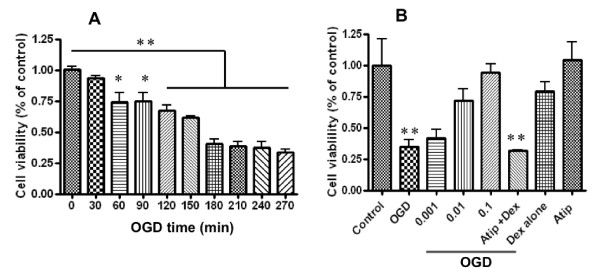
**Renoprotective effect of dexmedetomidine (Dex) *in vitro***. Cultured HK2 cell injury was provoked with culture media deprived of oxygen and glucose (OGD). (**A**) The time course of OGD. (**B**) The dose response of Dex (0.001 to 0.1 nM) against OGD induced injury in the presence or absence of α_2 _adrenoceptor antagonist, atipamazole (Atip) (1 nM). Data are expressed as mean ± SD (*n *= 5). *P < 0.05; **P < 0.01 vs zero time point or control.

To determine whether dexmedetomidine provides reno-protection *in vitro*, we exposed HK2 cells, a well-characterized human kidney proximal tubular cell line to oxygen and glucose deprivation (OGD), established to mimic the ischemic phase of renal ischemic reperfusion injury in our previous studies [26]. Cell viability analysis using MTT assay showed a time-dependent induction of injury with marked cell death (60% reduction in viability) occurring after 180-minutes OGD (0.39 ± 0.07 versus 1.0 ± 0.04 of control; *P *< 0.05) and was, therefore, used in subsequent experiments to determine the cytoprotective effects of dexmedetomidine (Figure 1A).

Incubating HK2 cells with dexmedetomidine (0.001 to 0.1 nM) before OGD exposure dose-dependently inhibited injury (Figure 1B). Treatment with 0.1 nM dexmedetomidine increased cell viability to 94% (from 0.35 ± 0.10 to 0.94 ± 0.12; *P *< 0.05), compared with the positive control (OGD). The cytoprotective effect of dexmedetomidine was abolished by co-treatment with the α2 adrenoceptor antagonist, atipamezole (1 nM), suggesting dexmedetomidine confers protection to renal epithelial cells *via *α_2 _adrenoceptor activation. Treatment of naïve controls with either dexmedetomidine (0.1 nM) or atipamezole (1 nM) did not significantly decrease cell viability (0.8 ± 0.13 and 1.0 ± 0.25 respectively versus control). Therefore, neither dexmedetomidine nor atipamezole were cytotoxic to HK2 cells.

**Figure 2:**
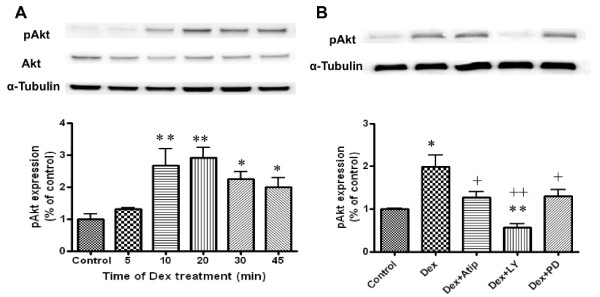
**Molecular mechanisms of renoprotection afforded by dexmedetomidine (Dex) *in vitro***. Cultured human kidney proximal tubular cells (HK2) cells were treated with dexmedetomidine and the time course of phospho-a serine/threonine protein kinase (pAkt) expression in culture homogenised supernatant was assessed with western blot (**A**) and its effect was abolished with the highly selective inhibitor of PI3-Akt, LY294002 (LY) (50 μM), but not by α2 adrenoceptor antagonist, atipamazole (Atip) (1 nM) and mitogen-activated protein kinase (MAPK), PD98059 (PD) (50 μM) (**B**). Data are expressed as the percentage of control (mean ± SD; *n *= 4). *P < 0.05; **P < 0.01 vs control; +*P *< 0.05; ++*P *< 0.01 vs Dex.

Activation of the Akt pathway plays a key role in cytoprotective signaling and has been demonstrated to ameliorate renal injury in IRI mice [29]. To determine whether dexmedetomidine activates Akt, we measured phosphorylated Akt (pAkt) levels in HK2 cells incubated with media containing 0.1 nM dexmedetomidine for 5, 10, 20, 30 and 45 minutes (Figure 2A). Dexmedetomidine induced significant increases in pAkt at all time-points; whereas, total Akt protein levels were not altered. The magnitude of Akt phosphorylation peaked after 20 minutes (2.91 ± 0.54 versus 1.0 ± 0.25 of control; *P *< 0.01) and was used in subsequent experiments to identify upstream mediators of Akt activation. Atipamezole reduced, but could not completely abolish dexmedetomidine-induced phosphorylation of Akt, suggesting that pAkt activation may be partially dependent on α_2 _adrenoceptors (Figure 2B). LY294002 and PD98059 have been shown to act *in vivo *as highly selective inhibitors of PI3-Akt and mitogen-activated protein kinase (MAPK) cascades respectively. LY294002 (50 μM) rather than PD98059 (50 μM) completely abolished dexmedetomidine-induced activation of Akt (0.6 ± 0.2 versus 1.7 ± 0.2 of Dex only; *P *< 0.01), indicating that pAkt was generated in a PI3K-dependent manner.

### Dexmedetomidine reduces IRI pathological changes *in vivo*

**Figure 3:**
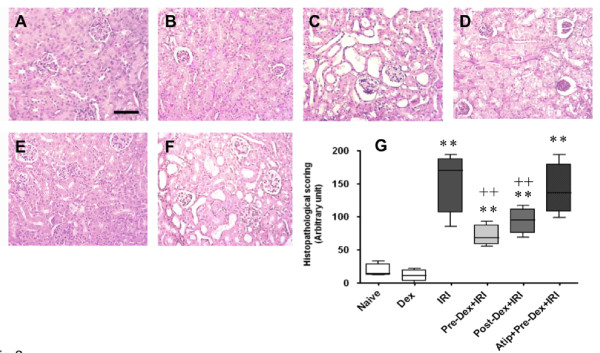
***In vivo *renoprotection by dexmedetomidine (Dex) vs renal ischemia-reperfusion injury (IRI)-histology**. Adult male mice were pretreated or post-treated with dexmedetomidine alone or in combination with α_2 _adrenoceptor antagonist, atipamazole (Atip) (1 nM) followed by clamping of the bilateral renal pedicle for 25 minutes. Reno-protection of Dex was assessed histologically (H&E staining). Representative microphotographs were taken from a naïve control (**A**), Dex alone (**B**), IRI alone (**C**), pre-Dex treatment (25 μ/kg) +IRI (**D**), post-Dex treatment (25 microgm/kg) + IRI (**E**) and Atip + pre-Dex+IRI (**F**). (**G**) Quantification of histological scoring following IRI in mice pretreated as above. Bar = 100 μm. Data are mean ± SD (*n *= 5). **P < 0.01 vs control. ++*P *< 0.01 vs IRI.

Next, we investigated whether dexmedetomidine provides protection against renal IRI in mice when the renal pedicle of both kidneys are clamped for 25 minutes. Twenty-four hours after IRI, kidneys and blood were harvested for histological assessment and for renal function (serum creatinine and urea), respectively. Histopathological assessment of cortical tubular damage was conducted by an investigator blinded to the experimental protocol. IRI significantly increased histopathological scoring (151 ± 45 versus 19 ± 9 of naive control; *P *< 0.01), as illustrated by severe tubular lumen dilatation, flattened renal epithelial cells and loss of nuclear staining (Figure 3C). Pre- and post-treatment with dexmedetomidine (25 μg/kg) resulted in a 53% and 38% reduction in damage (72 ± 15; *P *< 0.01 and 94 ± 19; *P *< 0.05) compared with IRI group, respectively (Figure 3G). Atipamazole (250 μg/kg) given prior to dexmedetomidine pre-treatment significantly inhibited the reno-protective action (142 ± 38; *P *< 0.01). Treatment of naïve animals with dexmedetomidine had no deleterious effects on kidney tissue (11 ± 9 versus 19 ± 9 of naïve control), in agreement with *in vitro *findings.

**Figure 4:**
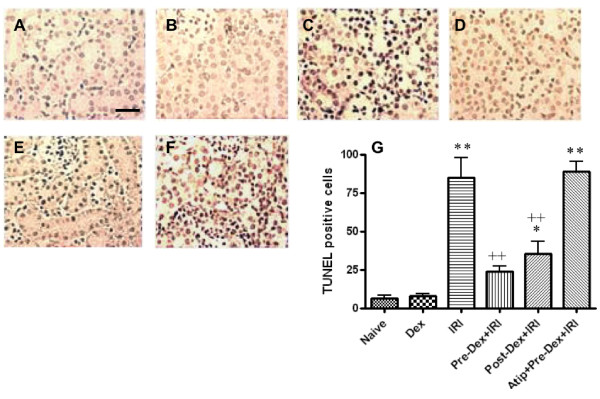
***In vivo *reno-protection afforded by dexmedetomidine (Dex) vs renal ischemia-reperfusion injury (IRI)- TUNEL staining**. Adult male mice were pretreated or post-treated with Dex alone or in combination with α_2 _adrenoceptor antagonist, atipamazole (Atip) (1 nM) followed by clamping the bilateral renal pedicle for 25 minutes and the renoprotection of Dex was assessed with terminal deoxynucleotidyl transferase dUTP nick end (TUNEL) staining. Representative microphotographs taken from a naïve control (**A**), Dex alone (**B**), IRI alone (**C**), pre-Dex treatment (25 μg/kg) +IRI (**D**), post-Dex treatment (25 μg/kg) + IRI (**E**) and Atip + pre-Dex+IRI (**F**). (**G**) Quantification of TUNEL positive cell counting following IRI in mice pretreated as above. Bar = 100 μm. Data are mean ± SD (*n *= 5 to 6). *P < 0.05, **P < 0.01 vs control. ++*P *< 0.01 vs IRI.

To evaluate kidney damage at the cellular level, we used terminal deoxynucleotidyl transferase-mediated digoxigenindeoxyuridine nick-end labeling (TUNEL) staining to detect dead tubular cells. IRI significantly increased TUNEL-positive cells (85.0 ± 28.5 versus 6.2 ± 5.3 of naive control, *P *< 0.01). Pre- and post-treatment with dexmedetomidine resulted in a 72% and 58% reduction in cell death (23.8 ± 7.5 and 35.2 ± 18.2; both *P *< 0.01), compared to IRI mice, respectively (Figure 4). Cellular renoprotection was abolished when dexmedetomidine pre-treatment was preceded by atipamazole (89.0 ± 14.3; *P *< 0.01). No difference in TUNEL staining was seen when dexmedetomidine was given to mice not subjected to renal injury (8.0 ± 2.3; NS versus naïve controls).

**Figure 5:**
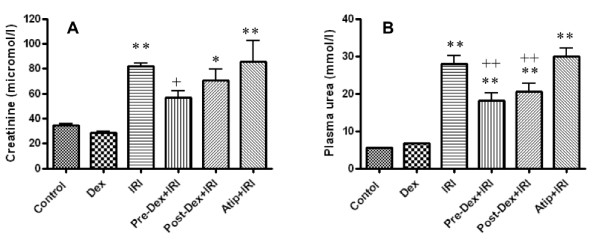
***In vivo *renoprotection afforded by dexmedetomidine (Dex) vs renal ischemia-reperfusion injury (IRI)**. Adult male Mice were pretreated or post-treated with Dex alone or in combination with α_2 _adrenoceptor antagonist, atipamazole (Atip) (1 nM) and the renoprotection of Dex was assessed with plasma creatinine (**A**) and urea measurement (**B**). Data are mean ± SD (*n *= 5 to 6). * *P *< 0.05, **P < 0.01 vs control. +*P *< 0.05, ++*P *< 0.01 vs IRI.

A similar pattern of changes were noted in renal function. After IRI, the plasma creatinine rose from 34.6 ± 2.2 to 81.8 ± 6.4 μM/L. Pre-treatment with dexmedetomidine was protective, with creatinine showing no significant difference from animals not subjected to renal injury. When dexmedetomidine pre-treatment was preceded by atipamazole, this abrogated the protective effect so that creatinine was significantly higher (85.8 ± 37.8; *P *< 0.01) than that seen in the uninjured animals (Figure 5A). Similarly, IRI-induced increase in plasma urea (from 5.4 ± 0.2 to 27.9 ± 5.0 mM/L) was significantly reduced following pre-treatment with dexmedetomidine (18.22 ± 4.3; *P *< 0.05), an effect that was completed abolished by atipamazole (30.0 ± 4.7; *P *< 0.01) (Figure 5B).

### Dexmedetomidine attenuates Toll-like Receptor 4 (TLR4) expression in tubular cells

**Figure 6:**
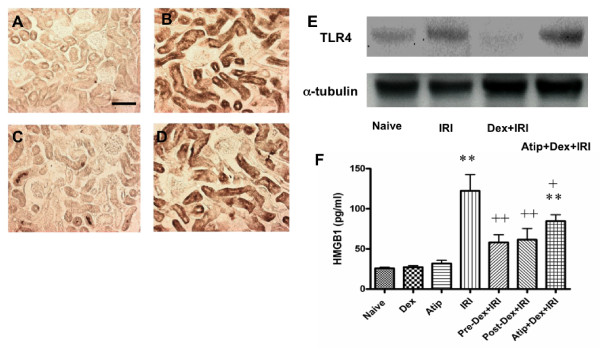
**Anti-inflammatory effect afforded by dexmedetomidine (Dex) *in vivo***. Toll-like receptor 4 (TLR4) expression was assessed with *in situ *immunostaining and Western blot. Naïve control (**A**); Renal ischemia-reperfusion injury (IRI) (**B**); Dex+IRI (**C**); Atip (atipamazole) + pre-Dex+IRI (**D**) and Western blot (**E**). (**F**) Plasma level of High-mobility group protein B1 (HMGB1). Bar = 100 μm. Data are mean ± SD (*n *= 4). **P < 0.01 vs control. +*P *< 0.05, ++*P *< 0.01 vs IRI.

To investigate the molecular mechanisms of dexmedetomidine-induced renoprotection, we assessed toll-like receptor 4 (TLR4) expression *in situ *and its upstream mediator, high mobility group box-1 (HMGB1) nuclear protein in plasma. HMGB1 and TLR4 signaling play a pivotal role in the coordination of inflammatory responses in renal IRI [30,31]. There was no detectable staining when primary antibody was omitted (data not shown) while TLR4 expression was at very minimal level in the naïve control (Figure 6A). However, marked increases in renal TLR4 expression were detected in IRI mice using *in situ *immunostaining (Figure 6B) and Western blot (Figure 6E). Pre-treatment with dexmedetomidine resulted in a dramatic decrease in TLR4 expression (Figure 6C) to a level even lower than that of control (Figure 6E), which was effectively restored to even the higher level than that in the control by atipamazole (Figure 6D). Similarly, plasma HMGB1 levels were dramatically elevated in IRI mice (from 25.9 ± 2.9 to 122.4 ± 40.3 pg/ml; *P *< 0.001), compared to control. Pre- and post-treatment with dexmedetomidine significantly attenuated the rise in HMGB1 (58.1 ± 19.5 and 61.5 ± 28.3; both *P *< .01) compared to IRI mice, respectively (Figure 6F). The protective effects of dexmedetomidine pre-treatment on HMGB1 upregulation were partially inhibited by atipamazole (61.5 ± 28.3; *P *< 0.05) relative to IRI mice. Together, these findings suggest that HMGB1 and TLR4 pro-inflammatory signaling in renal IRI may be partially dependent on an α2 adrenoceptor-mediated mechanism. No change was seen in naïve mice treated with dexmedetomidine.

### Dexmedetomidine protects from renal failure

**Figure 7:**
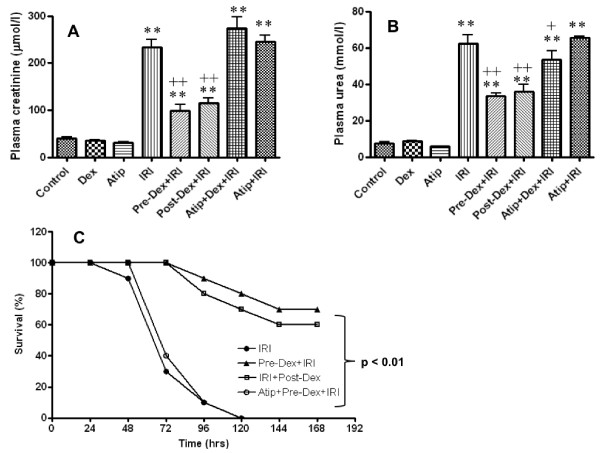
**Renal failure induced by ischemia-reperfusion prevented by dexmedetomidine (Dex)**. Mice received Dex before or after IRI and kidney injury was induced by clamping the right renal pedicle for 40 minutes while the contralateral kidney was removed. The animals were allowed for 24 hrs before functional measurements (**A **- Creatinine; **B **- Urea), or up to seven days for the long term Kaplan-Meier survival curve analysis (*n *= 10) (**C**). Atip = atipamazole; IRI = Ischemia reperfusion injury. Data are mean ± SD (*n *= 5). **P < 0.01 vs control. +*P *< 0.05, ++*P *< 0.01 vs IRI.

To assess whether dexmedetomidine was also effective in the context of a more severe insult to renal function, we performed additional experiments in which the right renal pedicle was clamped for 40 minutes and the left kidney was removed. The mean value of plasma creatinine and urea rose more than seven-fold at 24 h after IRI (to 233.5 ± 34.1 μM/L and 62.1 ± 10.2 mM/L, respectively). Administration of dexmedetomidine either before or after IRI significantly attenuated the rise in creatinine and urea values (both *P *< 0.01) relative to IRI controls (Figure 7A, B). Atipamezole treatment did not change creatinine and urea in IRI mice but significantly inhibited the renal protective effects of dexmedetomidine (*P *< 0.001, all comparisons). Long-term survival (no signs of illness at seven days) was noted in 70% and 60% of animals treated with dexmedetomidine before and after renal IRI. By contrast, animals not treated with dexmedetomidine or receiving atipamezole combined dexmedetomidine fared much worse. Within three days, 65% of these animals were dead (either spontaneously or killed because of severe ill health measured against previously reported predefined criteria [28] and no animals survived beyond five days after IRI (*P *< 0.001 versus pre- and post-dexmedetomidine groups; Figure 7C).

## Discussion

Our work showed that dexmedetomidine induced a sustained up-regulation of phospho-Akt and protected against oxygen-glucose deprivation in a human kidney cell line; its effects were blocked by atipamezole. *In vivo*, dexmedetomidine attenuated the HMGB-1/TLR-4 pathway, preserving tubular architecture and reducing cell death. Associated with the improved histological findings, the level of renal dysfunction was minimized and renal failure was prevented after severe IRI resulting in improved survival. The organ-protective effect was abolished with α_2 _adrenoreceptor antagonist, indicating that dexmedetomidine acted in an α_2 _adrenoreceptor dependent manner.

Our data demonstrated a significantly increased expression of phospho-Akt in cultured tubular cells after treatment with dexmedetomidine (Figure 2). This was blocked partially by an α_2 _adrenoceptor antagonist and significantly reduced by a PI3K inhibitor, indicating that the dexmedetomidine activates Akt *via *both α_2 _adrenoceptor dependent and independent-PI3K coupling. In summation, we consider it likely that the activation of PI3K-Akt is one of the survival cascades activated by dexmedetomidine to induce cytoprotection (Figure 1). The PI3K-Akt pathway promotes cell survival by phosphorylating the proapoptotic Bcl-2-associated death promotor (BAD) and up-regulating the expression of anti-apoptotic Bcl-2 and Bcl-xl [32], inhibiting the caspase-controlled intrinsic apoptotic pathway [33]. Consistent with our findings, dexmedetomidine has been shown to decrease the expression of pro-apoptotic factors such as caspase-3 [34] and Bax whilst increasing the expression of anti-apoptotic Bcl-2 and Mdm-2 [35] in the brain. Akt signaling has previously been shown to be critical to recovery from renal IRI injury and therefore, it can be concluded that dexmedetomidine protection may involve Akt signaling.

**Figure 8:**
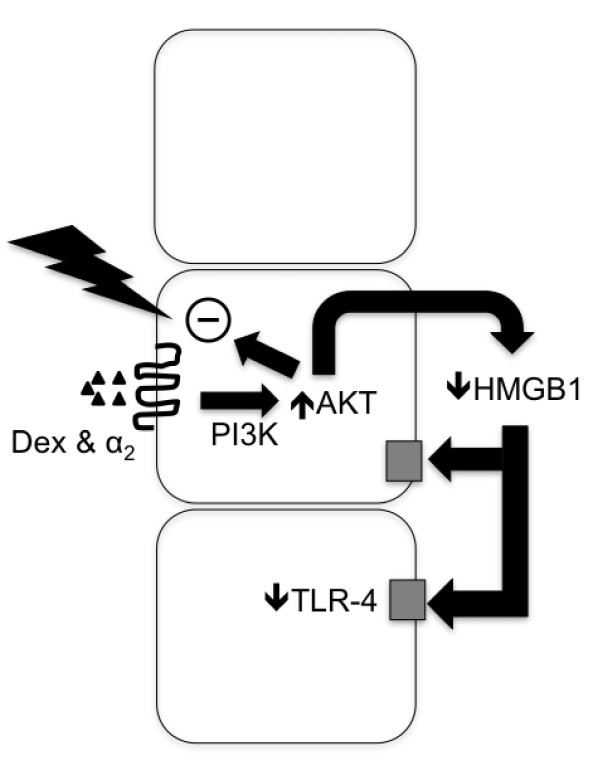
**Putative mechanism of dexmedetomidine (Dex) renoprotection**. Dexmedetomidine stimulates the α_2 _adrenoceptor (α_2_) to induce phosphoinositol-3-kinase (PI3K) phosphorylation of Akt (pAKT) and activation of the downstream cell survival signaling to block ischemia induced injury. Ischemia is denoted by the "flash" symbol. Improved cellular survival leads to reduced release of the damage-associated molecular pattern High-mobility group protein B1 (HMGB1). Reduced HMGB1 decreases the inflammatory drive that both increases Toll-like receptor 4 (TLR4) expression and activates the detrimental effect of TLR4 signaling in renal ischemia. Co-receptors for HMGB1 include TLRs and Interleukin-1 (IL-1) receptor; when HMGB1 binds to the ligand (for example, lipopolysaccharide or IL-1) this augments subsequent proinflammatory signaling. Therefore, dexmedetomidine acts proximally in the pathway (at the cytoprotective level) to prevent the elaboration of renal ischemic injury. 'Lightening bolt' denotes ischemia. 'Square' denotes HMGB1 co-receptor.

In addition to its cytoprotective effects, our studies demonstrated that dexmedetomidine suppressed the TLR-4 mediated inflammatory circuitry. Expression of TLR-4 has been shown to be triggered through endogenous ligands, including damage-associated molecular patterns (DAMPs) and cytokines. High mobility group box 1 (HMGB-1) is a potent DAMP released from dying cells during tissue ischemia. It binds to TLR-4 initiating down-stream NF-κB signaling cascade [36] substantially augmenting the synthesis of pro-inflammatory cytokines such as TNF-α and IL-1β [37]. Recent work by Wu *et al*., [38] demonstrated that TLR4-deficient or the adaptor molecule myD88-deficient mice were protected from both kidney dysfunction and histological damage induced by renal IRI. Generation of pro-inflammatory cytokine (IL-6, IL-1β, and TNF-α) and chemokines (MIP-2 and MCP-1) was inhibited, together with a parallel decline of macrophage and neutrophil infiltration. Pre-treatment of dexmedetomine resulted in almost complete attenuation of TLR-4 expression associated with decreased cell death of tubular epithelial cells. We propose that dexmedetomidine may have prevented the increased expression of TLR-4 by attenuating tissue injury (evidenced by lower systemic HMGB1 levels and reduced TUNEL staining) and through co-existent anti-inflammatory actions [39-41] (Figure 8). Herein, we did not further explore the well described anti-inflammatory effects of dexmedetomidine (beyond HMGB1 levels); however, dexmedetomidine reduces systemic levels of IL-6 and TNF-α following lipopolysaccharide infusion in rats [39] and following cardiac surgery [40] and sepsis in humans [42]. It is, therefore, plausible that the anti-inflammatory actions of dexmedetomidine contributed to the reduced TLR-4 expression following renal ischemia.

Our study showed a marked improvement in renal morphology and function with reduced nitrogenous waste accumulation following treatment of dexmedetomidine. This protection was attenuated by atipamezole, an α_2 _adrenoreceptor antagonist, confirming dependence on α_2_-adrenoceptor agonism. Similarly dexmedetomidine's neuroprotective effect is mediated by α_2_-adrenoceptor signaling [19]. Consistent with evidence from neuroprotection, our *in vitro *data suggest that the primary effect of dexmedetomidine is cytoprotection; nonetheless, *in vivo *it is likely that improved renal blood flow may have contributed to improved renal function and recovery from ischemia. Indeed modulation of vasoreactivity, through reduced sympathetic drive, has been shown to be an important mechanism of α_2 _adrenoceptor agonist renoprotection [21,24,25]. In a model of radiocontrast nephropathy α_2_-adrenoceptor activation with dexmedetomidine resulted in improved renal function, an effect attributable to improved renal blood flow [21]. However, α_2_-adrenoceptor activation was not associated with cytoprotection from radiocontrast exposure *in vitro *[21] indicating that there are differing mechanisms of radiocontrast and ischemic injury in the kidney. The local responses to α_2_-adrenoceptor activation in the kidney include vasodilatation [21], inhibition of renin release, increased glomerular filtration and increased secretion of sodium and water [43,44]. α_2_-adrenoceptor agonists may preserve glomerular filtration by preventing reduced renal blood flow following reperfusion associated vasospasm. They may also provoke diuresis by opposing the activity of arginine vasopressin in the collecting duct as well reducing aquaporin expression [45]. In combination, cytoprotection, improved glomerular filtration and diuretic actions may have improved renal function following ischemic injury.

α_2_-adrenoceptor agonists have diverse utility in the perioperative period; their renoprotective qualities are complemented by their analgesic qualities that reduce the necessity of other analgesics. Reduced use of non-steroidal anti-inflammatory drugs and opioids may be of particular interest as non-steroidal anti-inflammatory drugs increase the risk of AKI and opioids accumulate in AKI. Furthermore, the hemodynamic control, cardioprotection and mild diuretic properties of α_2_-adrenoceptor agonists may indirectly support renal function. We consider there are multiple reasons to consider a large prospective randomized controlled trial of the reno-protective qualities of α_2_-adrenoceptor agonists.

## Conclusions

AKI associated with ischemia-reperfusion injury is a significant contributor to morbidity and mortality in the perioperative period. dexmedetomidine provides renoprotection against renal IRI whether given as a prophylactic or therapeutic measure. Our data complement preliminary clinical data showing that prophylactic clonidine treatment prevented renal dysfunction attributable to cardiac surgery and that dexmedetomidine improved renal function following thoracic surgery. If our data can be extrapolated to clinical settings, dexmedetomidine may prove protective against IRI associated acute renal failure.

## Key messages

• Acute kidney injury following surgery significantly increases mortality with no proven preventative therapy.

• The α_2 _adrenoceptor agonist dexmedetomidine has organoprotective properties.

• Dexmedetomidine activates cell survival signal pAKT *via *α_2 _adrenoceptors to reduce cell death and HMGB1 release and subsequently inhibits TLR4 signaling to provide renoprotection.

• Dexmedetomidine may have both cytoprotective and anti-inflammatory effects to protect against renal injury following ischemia-reperfusion.

• If our data can be extrapolated to clinical settings, dexmedetomidine may prove protective against IRI associated acute renal failure.

## Abbreviations

AKI: acute kidney injury; AKT: a serine/threonine protein kinase; DAMPs: damage-associated molecular patterns; Dex: dexmedetomidine; HK2: human kidney proximal tubular cells; HMGB-1: high-mobility group protein B1; IL: interleukin; IRI: ischemia-reperfusion injury; MTT: 3- (4,5-Dimethyl-2-thiazolyl) -2, 5-diphenyl -2H- tetrazolium bromide; NFκB: nuclear factor kappa-light-chain-enhancer of activated B cells; OGD: oxygen glucose deprivation; PI3K: phosphatidylinositol 3-kinase; TLR-4: toll-like receptor 4; TNF-α: tumor necrosis factor α; TUNEL: terminal deoxynucleotidyl transferase dUTP nick end labeling.

## Competing interests

MM has been a consultant for Abbott Laboratories, Abbott Park, IL, USA, to facilitate registration of dexmedetomidine in the United States. There is no conflict of interest involving the other authors.

## Authors' contributions

JG carried out the *in vivo *and *in vitro *studies and drafted the manuscript. PS carried out the *in vivo *studies and drafted the manuscript. HZ, HRW and RDS drafted the manuscript. NT carried out ELISA measurements. PX and MM participated in the design of the study. DM designed studies, analysed data and drafted the manuscript. All authors read and approved the final manuscript.

## References

[B1] Perico N Cattaneo D Sayegh MH Remuzzi G Delayed graft function in kidney transplantation Lancet 2004 364 1814 1827 10.1016/S0140-6736(04)17406-0 15541456

[B2] Stafford-Smith M Shaw A Swaminathan M Cardiac surgery and acute kidney injury: emerging concepts Curr Opin Crit Care 2009 15 498 502 10.1097/MCC.0b013e328332f753 19812485

[B3] Koo DD Welsh KI Roake JA Morris PJ Fuggle SV Ischemia/reperfusion injury in human kidney transplantation: an immunohistochemical analysis of changes after reperfusion Am J Pathol 1998 153 557 566 10.1016/S0002-9440(10)65598-8 9708815 PMC1852972

[B4] Kashyap VS Cambria RP Davison JK L'Italien GJ Renal failure after thoracoabdominal aortic surgery J Vasc Surg 1997 26 949 955 10.1016/S0741-5214(97)70006-5 9423709

[B5] Kupiec-Weglinski JW Busuttil RW Ischemia and reperfusion injury in liver transplantation Transplant Proc 2005 37 1653 1656 10.1016/j.transproceed.2005.03.134 15919422

[B6] Sear JW Kidney dysfunction in the postoperative period Br J Anaesth 2005 95 20 32 10.1093/bja/aei018 15531622

[B7] Borthwick E Ferguson A Perioperative acute kidney injury: risk factors, recognition, management, and outcomes BMJ 2010 341 c3365 10.1136/bmj.c3365 20603317

[B8] Englberger L Suri RM Greason KL Burkhart HM Sundt TM Daly RC Schaff HV Deep hypothermic circulatory arrest is not a risk factor for acute kidney injury in thoracic aortic surgery J Thorac Cardiovasc Surg 2011 141 552 558 10.1016/j.jtcvs.2010.02.045 20392457

[B9] Cerda J Lameire N Eggers P Pannu N Uchino S Wang H Bagga A Levin A Epidemiology of acute kidney injury Clin J Am Soc Nephrol 2008 3 881 886 10.2215/CJN.04961107 18216347

[B10] Schrier RW Wang W Poole B Mitra A Acute renal failure: definitions, diagnosis, pathogenesis, and therapy J Clin Invest 2004 114 5 14 15232604 10.1172/JCI22353 PMC437979

[B11] Carden DL Granger DN Pathophysiology of ischaemia-reperfusion injury J Pathol 2000 190 255 266 10.1002/(SICI)1096-9896(200002)190:3<255::AID-PATH526>3.0.CO;2-6 10685060

[B12] Gluba A Banach M Hannam S Mikhailidis DP Sakowicz A Rysz J The role of Toll-like receptors in renal diseases Nat Rev Nephrol 2010 6 224 235 10.1038/nrneph.2010.16 20177402

[B13] Arslan F Keogh B McGuirk P Parker AE TLR2 and TLR4 in ischemia reperfusion injury Mediators Inflamm 2010 2010 704202 20628516 10.1155/2010/704202 PMC2902053

[B14] Lotze MT Tracey KJ High-mobility group box 1 protein (HMGB1): nuclear weapon in the immune arsenal Nat Rev Immunol 2005 5 331 342 10.1038/nri1594 15803152

[B15] Yu M Wang H Ding A Golenbock DT Latz E Czura CJ Fenton MJ Tracey KJ Yang H HMGB1 signals through toll-like receptor (TLR) 4 and TLR2 Shock 2006 26 174 179 10.1097/01.shk.0000225404.51320.82 16878026

[B16] Hoste EA Kellum JA Incidence, classification, and outcomes of acute kidney injury Contrib Nephrol 2007 156 32 38 17464113 10.1159/000102013

[B17] Hall JE Uhrich TD Barney JA Arain SR Ebert TJ Sedative, amnestic, and analgesic properties of small-dose dexmedetomidine infusions Anesth Analg 2000 90 699 705 10.1097/00000539-200003000-00035 10702460

[B18] Sanders RD Maze M Alpha2-adrenoceptor agonists Curr Opin Investig Drugs 2007 8 25 33 17263182

[B19] Ma D Hossain M Rajakumaraswamy N Arshad M Sanders RD Franks NP Maze M Dexmedetomidine produces its neuroprotective effect via the alpha 2A-adrenoceptor subtype Eur J Pharmacol 2004 502 87 97 10.1016/j.ejphar.2004.08.044 15464093

[B20] Kuhmonen J Pokorny J Miettinen R Haapalinna A Jolkkonen J Riekkinen P Sr Sivenius J Neuroprotective effects of dexmedetomidine in the gerbil hippocampus after transient global ischemia Anesthesiology 1997 87 371 377 10.1097/00000542-199708000-00025 9286902

[B21] Billings FTt Chen SW Kim M Park SW Song JH Wang S Herman J D'Agati V Lee HT alpha2-Adrenergic agonists protect against radiocontrast-induced nephropathy in mice Am J Physiol Renal Physiol 2008 295 F741 748 10.1152/ajprenal.90244.2008 18579700

[B22] Frumento RJ Logginidou HG Wahlander S Wagener G Playford HR Sladen RN Dexmedetomidine infusion is associated with enhanced renal function after thoracic surgery J Clin Anesth 2006 18 422 426 10.1016/j.jclinane.2006.02.005 16980158

[B23] Kulka PJ Tryba M Zenz M Preoperative alpha2-adrenergic receptor agonists prevent the deterioration of renal function after cardiac surgery: results of a randomized, controlled trial Crit Care Med 1996 24 947 952 10.1097/00003246-199606000-00012 8681596

[B24] Solez K Ideura T Silvia CB Hamilton B Saito H Clonidine after renal ischemia to lessen acute renal failure and microvascular damage Kidney Int 1980 18 309 322 10.1038/ki.1980.141 7463946

[B25] Tsutsui H Sugiura T Hayashi K Ohkita M Takaoka M Yukimura T Matsumura Y Moxonidine prevents ischemia/reperfusion-induced renal injury in rats Eur J Pharmacol 2009 603 73 78 10.1016/j.ejphar.2008.12.012 19101535

[B26] Rizvi M Jawad N Li Y Vizcaychipi MP Maze M Ma D Effect of noble gases on oxygen and glucose deprived injury in human tubular kidney cells Exp Biol Med (Maywood) 235 886 891 20472713 10.1258/ebm.2010.009366

[B27] Sanders RD Xu J Shu Y Januszewski A Halder S Fidalgo A Sun P Hossain M Ma D Maze M Dexmedetomidine attenuates isoflurane-induced neurocognitive impairment in neonatal rats Anesthesiology 2009 110 1077 1085 10.1097/ALN.0b013e31819daedd 19352168

[B28] Ma D Lim T Xu J Tang H Wan Y Zhao H Hossain M Maxwell PH Maze M Xenon preconditioning protects against renal ischemic-reperfusion injury via HIF-1alpha activation J Am Soc Nephrol 2009 20 713 720 10.1681/ASN.2008070712 19144758 PMC2663824

[B29] Satake A Takaoka M Nishikawa M Yuba M Shibata Y Okumura K Kitano K Tsutsui H Fujii K Kobuchi S Ohkita M Matsumura Y Protective effect of 17beta-estradiol on ischemic acute renal failure through the PI3K/Akt/eNOS pathway Kidney Int 2008 73 308 317 10.1038/sj.ki.5002690 18004295

[B30] Klune JR Dhupar R Cardinal J Billiar TR Tsung A HMGB1: endogenous danger signaling Mol Med 2008 14 476 484 18431461 10.2119/2008-00034.Klune PMC2323334

[B31] Franks NP Lieb WR Volatile general anaesthetics activate a novel neuronal K+ current Nature 1988 333 662 664 10.1038/333662a0 2453807

[B32] Datta SR Dudek H Tao X Masters S Fu H Gotoh Y Greenberg ME Akt phosphorylation of BAD couples survival signals to the cell-intrinsic death machinery Cell 1997 91 231 241 10.1016/S0092-8674(00)80405-5 9346240

[B33] Hennessy BT Smith DL Ram PT Lu Y Mills GB Exploiting the PI3K/AKT pathway for cancer drug discovery Nat Rev Drug Discov 2005 4 988 1004 10.1038/nrd1902 16341064

[B34] Dahmani S Rouelle D Gressens P Mantz J Effects of dexmedetomidine on hippocampal focal adhesion kinase tyrosine phosphorylation in physiologic and ischemic conditions Anesthesiology 2005 103 969 977 10.1097/00000542-200511000-00011 16249671

[B35] Engelhard K Werner C Eberspacher E Bachl M Blobner M Hildt E Hutzler P Kochs E The effect of the alpha 2-agonist dexmedetomidine and the N-methyl-D-aspartate antagonist S(+)-ketamine on the expression of apoptosis-regulating proteins after incomplete cerebral ischemia and reperfusion in rats Anesth Analg 2003 96 524 531 12538207 10.1097/00000539-200302000-00041

[B36] O'Neill LA Bowie AG The family of five: TIR-domain-containing adaptors in Toll-like receptor signalling Nat Rev Immunol 2007 7 353 364 10.1038/nri2079 17457343

[B37] Akira S Takeda K Kaisho T Toll-like receptors: critical proteins linking innate and acquired immunity Nat Immunol 2001 2 675 680 10.1038/90609 11477402

[B38] Wu H Chen G Wyburn KR Yin J Bertolino P Eris JM Alexander SI Sharland AF Chadban SJ TLR4 activation mediates kidney ischemia/reperfusion injury J Clin Invest 2007 117 2847 2859 10.1172/JCI31008 17853945 PMC1974864

[B39] Taniguchi T Kidani Y Kanakura H Takemoto Y Yamamoto K Effects of dexmedetomidine on mortality rate and inflammatory responses to endotoxin-induced shock in rats Crit Care Med 2004 32 1322 1326 10.1097/01.CCM.0000128579.84228.2A 15187514

[B40] Venn RM Bryant A Hall GM Grounds RM Effects of dexmedetomidine on adrenocortical function, and the cardiovascular, endocrine and inflammatory responses in post-operative patients needing sedation in the intensive care unit Br J Anaesth 2001 86 650 656 10.1093/bja/86.5.650 11575340

[B41] Sanders RD Hussell T Maze M Sedation & immunomodulation Crit Care Clin 2009 25 551 570 10.1016/j.ccc.2009.05.001 19576530

[B42] Memis D Hekimoglu S Vatan I Yandim T Yuksel M Sut N Effects of midazolam and dexmedetomidine on inflammatory responses and gastric intramucosal pH to sepsis, in critically ill patients Br J Anaesth 2007 98 550 552 10.1093/bja/aem017 17363413

[B43] Gertler R Brown HC Mitchell DH Silvius EN Dexmedetomidine: a novel sedative-analgesic agent Proc (Bayl Univ Med Cent) 2001 14 13 21 16369581 10.1080/08998280.2001.11927725 PMC1291306

[B44] Philipp M Brede M Hein L Physiological significance of alpha(2)-adrenergic receptor subtype diversity: one receptor is not enough Am J Physiol Regul Integr Comp Physiol 2002 283 R287 295 12121839 10.1152/ajpregu.00123.2002

[B45] Junaid A Cui L Penner SB Smyth DD Regulation of aquaporin-2 expression by the alpha(2)-adrenoceptor agonist clonidine in the rat J Pharmacol Exp Ther 1999 291 920 923 10525117

